# Influence Mechanism of Quantization Error on the Key Parameters of the Whole-Angle Hemisphere Resonator Gyroscope

**DOI:** 10.3390/mi17010143

**Published:** 2026-01-22

**Authors:** Xiuyue Yan, Jingyu Li, Pengbo Xiao, Tao Xia, Xingyuan Tang, Yao Pan, Kaiyong Yang, Hui Luo

**Affiliations:** College of Advanced Interdisciplinary Studies, National University of Defense Technology, Changsha 410073, China; yanxiuyue24@163.com (X.Y.); xiaopengbo09@nudt.edu.cn (P.X.); xiat06@163.com (T.X.); tangxingyuan2016@163.com (X.T.); panyao08@nudt.edu.cn (Y.P.); luohui.luo@163.com (H.L.)

**Keywords:** whole-angle hemispherical resonator gyroscope, hardware detection and driving circuits, quantization error, influence mechanism

## Abstract

The whole-angle hemispherical resonator gyroscope (WA-HRG) is critical to high-precision attitude control and navigational positioning, boasting significant deployment potential in both highly dynamic inertial navigation systems and industrial instrumentation. This paper presents a mechanistic analysis of quantization error inherent to the HRG’s hardware detection and driving circuits, focusing specifically on its impact on parameter calculation and driving control in whole-angle mode. Furthermore, a simulation platform was constructed to verify and elucidate the correlations between the effects of quantization error and key resonator parameters, such as the major axis amplitude and the standing wave azimuth. Compared to existing HRG error studies which frame quantization error as isolated circuit noise, this work uniquely uncovers the azimuth-modulated periodic behavior of quantization error within the WA-HRG. It also formalizes a quantitative relationship between quantization error and the resonator’s key parameters, laying a critical theoretical foundation for suppressing quantization error and enhancing accuracy in high-performance WA-HRGs.

## 1. Introduction

As a core sensor in inertial navigation systems [1,2], the HRG exhibits notable advantages, including strong interference resistance, excellent stability and long operational lifespan [2,3,4]. Particularly in whole-angle mode, it demonstrates enhanced compatibility with high-dynamic application scenarios [5,6,7], establishing itself as the preferred gyroscope in inertial navigation systems [8,9,10]. The HRG has been widely applied in the fields of ocean, land, aviation and space [1,4,8,9,10].

However, the detection and driving circuits of the WA-HRG inevitably introduce various error sources while performing core functions such as analog-to-digital conversion and driving control [11]. These error sources are of two types: one is random errors caused by circuit thermal noise [12], quantization noise and ripple noise; the other is systematic errors such as gain asymmetry errors [3,13,14,15,16], phase errors [15,17] and nonlinear errors [7,18,19,20]. The errors caused by these sources will continue to accumulate during signal processing and closed-loop control; these errors not only affect the accuracy of the gyroscope output [14,21] but also compromise the process of vibration mode rotation modulation. Therefore, these errors have become a key bottleneck limiting further accuracy enhancements of WA-HRGs [15,21].

In recent years, researchers have identified and compensated for multiple errors in the hardware circuits of HRGs and achieved significant results [3,7,13,15,16,17,18,19], particularly in the calibration and compensation of gain asymmetry errors, phase errors and nonlinear errors. These are deterministic errors, which can be calibrated and compensated for in the laboratory. Conversely, random errors, such as quantization errors, require the establishment of a random error model for analysis. Therefore, there has been little research on the errors caused by random disturbances during circuit operation. The impact of these overlooked error sources on the accuracy of gyroscopes is becoming increasingly significant. In the limited research on random noise, scholars have mainly focused on the study of noise characteristics in capacitance–voltage (C/V) conversion circuits and power circuits. For example, reference [12] investigates the noise characteristics of C/V conversion circuits in the HRG and confirms that the charge amplifier exhibits excellent noise suppression capability near the resonance frequency. Its noise power spectral density is reduced by about two orders of magnitude compared to traditional transimpedance amplifiers, providing theoretical support for noise suppression in C/V conversion circuits. As an inherent error type in analog-to-digital conversion circuits, quantization error has merely been identified and preliminarily suppressed in the latest research in the field of HRGs. Reference [22] first derived the closed-loop transfer function of circuit noise and identified C/V conversion circuits and analog-to-digital conversion (ADC) circuits as the primary noise sources of HRGs. Reference [23] employed Allan variance to analyze the noise composition of the HRG, demonstrating that quantization noise dominates the overall noise of the gyroscope and suppressed it by adjusting the parameters of the signal detection circuits. It is evident that there is a lack of in-depth research on the influence mechanisms of quantization errors. At the same time, methods to suppress quantization errors are relatively limited and lack a deep integration with the core parameters of HRGs. Reference [24] conducted research on the DAC quantization error of honeycomb-type MEMS gyroscopes and its impact on gyroscope output. The authors also proposed a suppression method based on perturbation signals, reducing bias instability from 0.060°/h to 0.0061°/h. Compared to MEMS gyroscopes, where mechanical noise is the dominant factor and quantization noise is secondary, the primary noise in HRGs is quantization noise, which is closely linked to the properties of the resonator and requires the design of dedicated suppression methods based on the resonator’s core parameters.

To address the above gap in the literature, this paper first conducts an analysis of quantization error in the detection and driving system of the WA-HRG, and then establishes corresponding mathematical models to investigate the specific effects on detection accuracy and driving control performance. Subsequently, a closed-loop simulation system for the WA-HRG is constructed based on the fundamental dynamics model of the HRG and the developed quantization error model. Finally, using the simulation platform, the relationships between the effects induced by quantization error and key parameters such as the major amplitude, the standing wave azimuth and ADC specifications are further simulated and validated. Through combined theoretical and simulation analysis, this work elucidates the specific influences of quantization error in the WA-HRG and clarifies their correlation with core performance parameters. This study holds significant theoretical and practical value for suppressing quantization error and enhancing the accuracy of WA-HRGs.

## 2. Fundamental Principles of Measurement and Control and Analysis of Quantization Error

### 2.1. Measurement Principle of the WA-HRG

**Figure 1:**
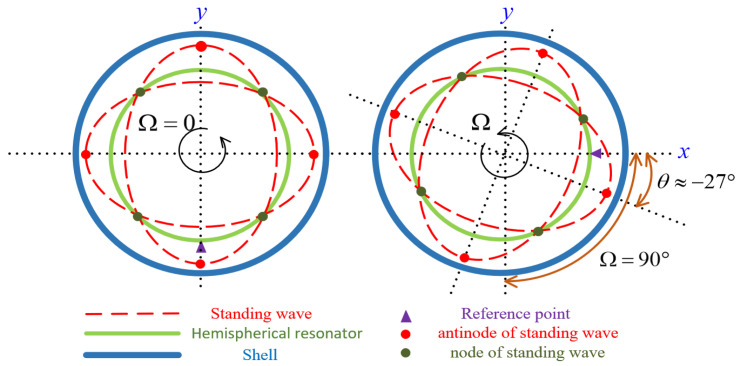
Schematic diagram of the standing wave precession.

Under ideal conditions, the positions of the standing wave’s antinodes and nodes of the hemispherical resonator remain constant in the absence of external angular rate input. When subjected to an external rotational excitation, the resonator of the WA-HRG generates standing wave precession due to the Coriolis effect, as illustrated in Figure 1.

**Figure 2:**
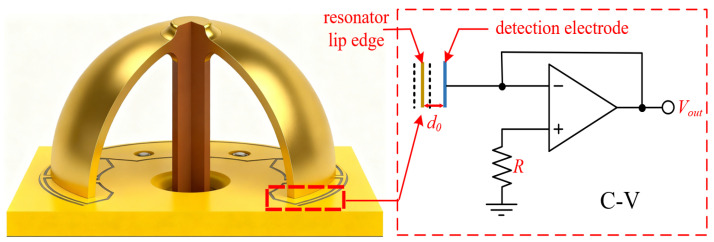
Schematic diagram of variable-spacing capacitive detection and C-V conversion.

At different positions of the standing wave azimuth, the vibration of the resonator induces variations in the capacitance between the resonator and the electrode plates [18]. The HRG system acquires displacement signals of the hemispherical resonator via a pair of detection electrodes (X and Y) positioned 45 degrees apart and converts these mechanical displacements into voltage signals, as illustrated in Figure 2.

These voltage signals are subsequently processed through demodulation and calculation algorithms to derive the standing wave azimuth θ. Then, the rotational angle or angular rate of the carrier is obtained based on the relationship between the standing wave azimuth θ and the external rotation angle Ω, as defined in (1), thereby achieving high-precision inertial measurement.

(1) θ=−γΩ

where γ is the precession factor. In practical HRG systems, γ typically ranges between 0.28 and 0.32 depending on resonator geometry and material properties.

### 2.2. Design of Measurement and Control Systems and Sources of Quantization Error

The HRG measurement and control system comprises the hemispherical resonator, the detection and driving circuits and the FPGA platform. In whole-angle mode, it primarily includes three control loops: the amplitude control loop, which supplements the energy attenuation of the amplitude due to damping, maintaining stable amplitude of the standing wave antinodes; the quadrature control loop, which suppresses the vibration of the wave nodes caused by frequency splitting; and the frequency control loop, which locks the resonant frequency near the eigenfrequency and generates a reference signal at the same frequency as the resonator for coherent demodulation algorithms.

**Figure 3:**
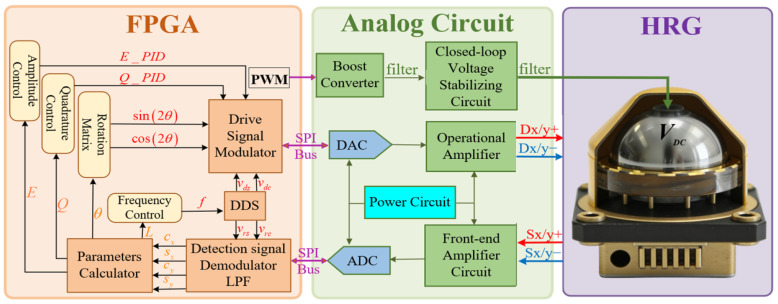
Measurement and control system of the HRG.

The control flowchart of the WA-HRG is illustrated in Figure 3. The vibration displacement signals from the interface between the hemispherical resonator lip and the detection electrodes are first converted into sensitive current signals via variable-gap capacitive detection technology. Then, these signals are processed by pre-amplification circuits. The processed signals are fed into an ADC in voltage form and converted into digital signals, which participate in the subsequent gyroscope parameter demodulation process within the FPGA platform. This stage introduces quantization error from the ADC.

Simultaneously, the vibration state of the hemispherical resonator requires precise regulation through driving forces. The gyroscope parameters from the FPGA platform and the digital control parameters output from the control loops are input into the driving signal modulation and synthesis module, which outputs the driving signals. Then, driving signals are sent to a Digital-to-Analog Converter (DAC) to be converted into analog driving voltage signals. The driving voltage signal is applied to the driving electrodes to achieve stable control of the resonator’s vibration. This stage introduces quantization error from the DAC.

### 2.3. Demodulation and Modulation Process of the WA-HRG

For an ideal hemispherical resonator operating in the second-order vibration mode, its Lissajous figure is a flat ellipse shape. In the elliptical coordinate system, the vibration displacement signals along the *x*-axis and *y*-axis directions can be expressed as (2)

(2) $$\left\{\begin{array}{c} V_{x}=k_{0}\left[a\cos2\theta \cos\left(\omega t+\varphi_{0}\right)-q\sin2\theta \sin\left(\omega t+\varphi_{0}\right)\right]\\ V_{y}=k_{0}\left[a\sin2\theta \cos\left(\omega t+\varphi_{0}\right)+q\cos2\theta \sin\left(\omega t+\varphi_{0}\right)\right] \end{array}\right.$$

where $V_{x},V_{y}$ are the voltage signals converted from the resonator’s vibration signals via the C/V detection circuit, $k_{0}$ represents the pre-amplification circuit gain, *a* represents the major axis amplitude of the ellipse, *q* represents the minor axis amplitude of the ellipse, θ represents the standing wave azimuth, and $\omega t+\varphi_{0}$ represents the vibration phase.

The voltage signals from (2), after ADC conversion, are input into the FPGA platform as digital quantities and demodulated with reference signals generated by a Direct Digital Synthesizer (DDS) that are at the same frequency as the resonator’s vibration displacement signals. The reference signals participating in demodulation can be expressed as (3):

(3) $$\left\{\begin{array}{c} v_{rc}=A\cos\left(\omega t+\varphi \right)\\ v_{rs}=A\sin\left(\omega t+\varphi \right) \end{array}\right.$$

where *A* represents the amplitude and ωt+φ represents the phase of the reference signal. The phase difference between the reference and detection signals is denoted as δ, i.e., $\delta =\varphi -\varphi_{0}$.

Multiplying the reference signals with the voltage signals respectively and filtering out the high-frequency components using an Infinite Impulse Response (IIR) digital filter yields the following expressions for $c_{x},s_{x},c_{y},s_{y}$ as (4):

(4) $$\left\{\begin{array}{c} \begin{array}{cc} c_{x} & =\frac{k_{0}A}{2}\left(a\cos2\theta \cos\delta +q\sin2\theta \sin\delta \right)\\ s_{x} & =\frac{k_{0}A}{2}\left(a\cos2\theta \sin\delta -q\sin2\theta \cos\delta \right)\\ c_{y} & =\frac{k_{0}A}{2}\left(a\sin2\theta \cos\delta -q\cos2\theta \sin\delta \right)\\ s_{y} & =\frac{k_{0}A}{2}\left(a\sin2\theta \sin\delta +q\cos2\theta \cos\delta \right) \end{array} \end{array}\right.$$

Further computation of $c_{x}$, $s_{x}$, $c_{y}$, $s_{y}$ yields the control parameters *E*, *Q*, *R*, *S*, *L* as (5):

(5) $$\left\{\begin{array}{cc} E & =c_{x}^{2}+s_{x}^{2}+c_{y}^{2}+s_{y}^{2}=\frac{k_{0}^{2}A^{2}}{4}\left(a^{2}+q^{2}\right)\\ Q & =2c_{x}s_{y}-2c_{y}s_{x}=\frac{k_{0}^{2}A^{2}}{2}aq\\ R & =c_{x}^{2}+s_{x}^{2}-c_{y}^{2}-s_{y}^{2}=\frac{k_{0}^{2}A^{2}}{4}\left(a^{2}-q^{2}\right)\cos4\theta \\ S & =2c_{x}c_{y}+2s_{x}s_{y}=\frac{k_{0}^{2}A^{2}}{4}\left(a^{2}-q^{2}\right)\sin4\theta \\ L & =2c_{x}s_{x}+2c_{y}s_{y}=\frac{k_{0}^{2}A^{2}}{4}\left(a^{2}-q^{2}\right)\sin2\delta \end{array}\right.$$

where $c_{x}$, $s_{x}$, $c_{y}$ and $s_{y}$ represent the in-phase and in-quadrature components along the *x*-axis and *y*-axis, respectively. The vibration energy *E* is kept as a constant $E_{0}$ through the amplitude-maintaining loop, the quadrature *Q* is suppressed by the quadrature control loop, and the variable *L* is used in the PI controllers of the Phase-Locked Loop (PLL). The pendulum variables *S* and *R* are utilized to estimate the orientation of the oscillation pattern.

Combining and computing the control parameter signals yields the expressions for the elliptical parameters (Equation (6)):

(6) $$\left\{\begin{array}{cc} a & =\frac{1}{2}\left(\sqrt{E+Q}+\sqrt{E-Q}\right)\\ q & =\frac{1}{2}\left(\sqrt{E+Q}-\sqrt{E-Q}\right)\\ \theta & =\frac{1}{4}\tan^{-1}\left(\frac{S}{R}\right)\\ \delta & =\frac{1}{2}\sin^{-1}\left(\frac{L}{\sqrt{E^{2}-Q^{2}}}\right) \end{array}\right.$$

Using the standing wave azimuth θ, a rotational transformation is applied to the control forces in the *x*- and *y*-axis directions to obtain the control force signals $f_{x}$, $f_{y}$, as shown in (7):

(7) $$\begin{array}{cc} \left[\begin{array}{c} f_{x}\\ f_{y} \end{array}\right] & =\left[\begin{array}{cc} \cos2\theta & -\sin2\theta \\ \sin2\theta & \cos2\theta \end{array}\right]\cdot \left[\begin{array}{c} F_{a}\\ F_{q} \end{array}\right]\\ \; & =\left[\begin{array}{c} \left\{\begin{array}{c} f_{as}\sin\left(\omega t+\varphi_{d}\right)\sin2\theta +f_{ac}\cos\left(\omega t+\varphi_{d}\right)\sin2\theta \\ +f_{qs}\sin\left(\omega t+\varphi_{d}\right)\cos2\theta +f_{qc}\cos\left(\omega t+\varphi_{d}\right)\cos2\theta \end{array}\right.\\ \left\{\begin{array}{c} f_{as}\sin\left(\omega t+\varphi_{d}\right)\sin2\theta +f_{ac}\cos\left(\omega t+\varphi_{d}\right)\sin2\theta \\ +f_{qs}\sin\left(\omega t+\varphi_{d}\right)\cos2\theta +f_{qc}\cos\left(\omega t+\varphi_{d}\right)\cos2\theta \end{array}\right. \end{array}\right] \end{array}$$

where $f_{as}$ is the amplitude control force, $f_{qc}$ is the quadrature suppression force, $f_{qs}$ is the virtual precession control force, $f_{ac}$ is the control force from the frequency control loop, and $\varphi_{d}$ represents the phase of the reference signal participating in modulation.

## 3. Modeling of Quantization Error

In the practical data acquisition process of the HRG detection and driving circuits, the use of ADC/DAC for conversion between continuous analog signals and discrete digital signals, as well as data transmission, inevitably introduces quantization error, which constrains fundamental performance metrics of the HRG such as angle random walk and bias stability.

### 3.1. Fundamental Theory of Quantization Error

**Figure 4:**
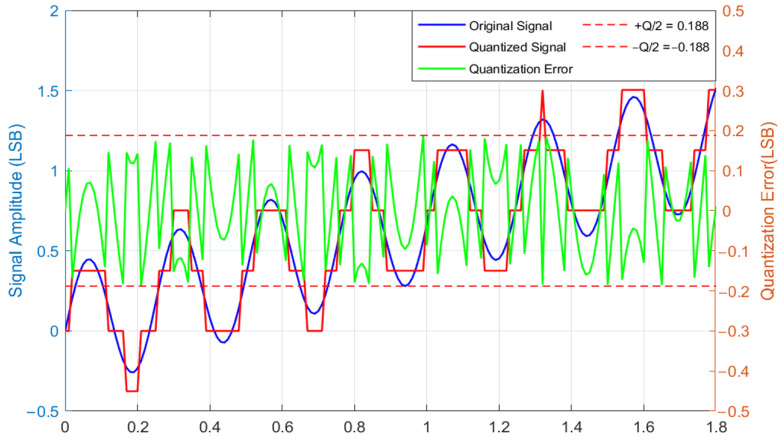
Signal quantization process and quantization error distribution.

Take uniform quantization as an example. In the process of uniform quantization, the quantization levels are distributed with equal spacing across the amplitude range $\left[x_{\min},x_{\max}\right]$. Given *n* quantization bits *n*, the total number of quantization levels is $N=2^{n}$, and the quantization step size is defined as $Q=\frac{x_{\max}-x_{\min}}{2^{n}-1}$. For a continuous input signal x(t), its quantized signal $\hat{x}\left(t\right)$ corresponds to the nearest quantization level. Quantization error is expressed as (8), with the error curve illustrated in Figure 4.

(8) $$e\left(t\right)=\hat{x}\left(t\right)-x\left(t\right),\;\left|e\left(t\right)\right|\leq \frac{Q}{2}$$

### 3.2. Standing Wave Azimuth and Angular Velocity Error

When quantization error is present, the actual voltage detection signals input to the FPGA platform for the *x*- and *y*-axis directions can be expressed as Equation (9):

(9) $$\left\{\begin{array}{c} V_{x,q}\left[n\right]=\mathrm{round}\left(\frac{V_{x}\left(t\right)}{\mathrm{LSB}}\right)\cdot \mathrm{LSB}=V_{x}\left(t\right)+e_{x}\left[n\right]\\ V_{y,q}\left[n\right]=\mathrm{round}\left(\frac{V_{y}\left(t\right)}{\mathrm{LSB}}\right)\cdot \mathrm{LSB}=V_{y}\left(t\right)+e_{y}\left[n\right] \end{array}\right.$$

where $e_{x}\left[n\right]$, $e_{y}\left[n\right]$ are quantization errors introduced in the *x*- and *y*-axis detection channels, $V_{x}$, $V_{y}$ are the ideal detection signals, and $V_{x,q}\left[n\right]$, $V_{y,q}\left[n\right]$ are the actual detection signals.

According to the demodulation process described in Section 2.3, the actual demodulated signals $c_{x,q}$, $s_{x,q}$, $c_{y,q}$, $s_{y,q}$ are expressed by Equations (10) to (13) respectively:

(10) $$\begin{array}{cc} c_{x,q} & =v_{rc}V_{x,q}\left[n\right]\\ \; & =k_{0}A\left[a\cos2\theta \cos\left(\omega t+\varphi_{0}\right)-q\sin2\theta \sin\left(\omega t+\varphi_{0}\right)+e_{x}\left[n\right]\right]\cos\left(\omega t+\varphi \right)\\ \; & \overset{\mathrm{LPF}}{\approx }\frac{k_{0}A}{2}\left(a\cos2\theta \cos\delta +q\sin2\theta \sin\delta \right)+k_{0}A\mathrm{LPF}\left[e_{x}\left[n\right]\cos\left(\omega t+\varphi \right)\right]\\ \; & =c_{x}+e_{cx} \end{array}$$

(11) $$\begin{array}{cc} s_{x,q} & =v_{rs}V_{x,q}\left[n\right]\\ \; & =k_{0}A\left[a\cos2\theta \cos\left(\omega t+\varphi_{0}\right)-q\sin2\theta \sin\left(\omega t+\varphi_{0}\right)+e_{x}\left[n\right]\right]\sin\left(\omega t+\varphi \right)\\ \; & \overset{\mathrm{LPF}}{\approx }\frac{k_{0}A}{2}\left(a\cos2\theta \sin\delta -q\sin2\theta \cos\delta \right)+k_{0}A\mathrm{LPF}\left[e_{x}\left[n\right]\sin\left(\omega t+\varphi \right)\right]\\ \; & =s_{x}+e_{sx} \end{array}$$

(12) $$\begin{array}{cc} c_{y,q} & =v_{rc}V_{y,q}\left[n\right]\\ \; & =k_{0}A\left[a\sin2\theta \cos\left(\omega t+\varphi_{0}\right)+q\cos2\theta \sin\left(\omega t+\varphi_{0}\right)+e_{y}\left[n\right]\right]\cos\left(\omega t+\varphi \right)\\ \; & \overset{\mathrm{LPF}}{\approx }\frac{k_{0}A}{2}\left(a\sin2\theta \cos\delta -q\cos2\theta \sin\delta \right)+k_{0}A\mathrm{LPF}\left[e_{y}\left[n\right]\cos\left(\omega t+\varphi \right)\right]\\ \; & =c_{y}+e_{cy} \end{array}$$

(13) $$\begin{array}{cc} s_{y,q} & =v_{rs}V_{y,q}\left[n\right]\\ \; & =k_{0}A\left[a\sin2\theta \cos\left(\omega t+\varphi_{0}\right)+q\cos2\theta \sin\left(\omega t+\varphi_{0}\right)+e_{y}\left[n\right]\right]\sin\left(\omega t+\varphi \right)\\ \; & \overset{\mathrm{LPF}}{\approx }\frac{k_{0}A}{2}\left(a\sin2\theta \sin\delta +q\cos2\theta \cos\delta \right)+k_{0}A\mathrm{LPF}\left[e_{y}\left[n\right]\sin\left(\omega t+\varphi \right)\right]\\ \; & =s_{y}+e_{sy} \end{array}$$

The secondary computed signals $R_{q}$, $S_{q}$ are expressed by Equations (14) and (15):

(14) $$\begin{array}{cc} R_{q} & ={\left(c_{x}+e_{cx}\right)}^{2}+{\left(s_{x}+e_{sx}\right)}^{2}-{\left(c_{y}+e_{cy}\right)}^{2}-{\left(s_{y}+e_{sy}\right)}^{2}\\ \; & \approx R+2(c_{x}e_{cx}+s_{x}e_{sx}-c_{y}e_{cy}-s_{y}e_{sy})\\ \; & =R+\Delta R \end{array}$$

(15) $$\begin{array}{cc} S_{q} & =2[\left(c_{x}+e_{cx}\right)\left(c_{y}+e_{cy}\right)+\left(s_{x}+e_{sx}\right)\left(s_{y}+e_{sy}\right)]\\ \; & \approx S+2(c_{x}e_{cy}+c_{y}e_{cx}+s_{x}e_{sy}+s_{y}e_{sx})\\ & =S+\Delta S \end{array}$$

According to Equations (6), (14) and (15), the quantized standing wave azimuth is expressed as Equation (16):

(16) $$\theta_{q}=\frac{1}{4}\arctan\left(\frac{S_{q}}{R_{q}}\right)=\frac{1}{4}\arctan\left(\frac{S+\Delta S}{R+\Delta R}\right)\approx \frac{1}{4}\arctan\left(\frac{S}{R}\right)+\frac{1}{4}\frac{R\Delta S-S\Delta R}{R^{2}+S^{2}}$$

where

(17) $$R^{2}+S^{2}=\frac{k_{0}^{4}A^{4}}{16}{\left(a^{2}-q^{2}\right)}^{2}$$

Therefore, the error of the standing wave azimuth Δθ and the error of angular velocity $\Delta \Omega_{z}$ are expressed as Equations (18) and (19), respectively:

(18) $$\begin{array}{cc} \Delta \theta & =\theta_{q}-\theta \approx \frac{4(R\Delta S-S\Delta R)}{k_{0}^{4}A^{4}{\left(a^{2}-q^{2}\right)}^{2}}\\ \; & \approx \frac{2\cos4\theta \cdot \left(c_{x}e_{cy}+c_{y}e_{cx}+s_{x}e_{sy}+s_{y}e_{sx}\right)-2\sin4\theta \cdot \left(c_{x}e_{cx}+s_{x}e_{sx}-c_{y}e_{cy}-s_{y}e_{sy}\right)}{k_{0}^{2}A^{2}\left(a^{2}-q^{2}\right)}\\ \; & \propto 2\cos4\theta \cdot e+2\sin4\theta \cdot e^{\prime } \end{array}$$

(19) $$\Delta \Omega_{z}=-{\dot{\theta }}_{q}/\gamma -\left(-\dot{\theta }/\gamma \right)=-\left(\dot{\theta }+\dot{\Delta }\theta \right)/\gamma -\left(-\dot{\theta }/\gamma \right)=-\dot{\Delta }\theta /\gamma$$

where $e_{cx},e_{sx},e_{cy},e_{sy},\Delta R,\Delta S$ are the errors introduced by quantization noise in different computation parameters, and $e,e^{\prime }$ are combined terms of quantization errors.

The derivation indicates that in the HRG, quantization error introduced by the ADC quantization process is modulated into azimuth-dependent azimuth and angular velocity errors. They exhibit a fourth harmonic distribution with the standing wave azimuth, forming periodic errors. Furthermore, these errors are strongly dependent on and suppressed by increasing ADC quantization bits and the major axis amplitude of the elliptical orbit. The error of the standing wave azimuth will cause the applied driving force to deviate from the actual vibration direction of the resonator, significantly reducing driving efficiency. Simultaneously, the unmatched angle introduces additional useless components, interfering with the stable vibration of the resonator and introducing driving force error.

Equations (18) and (19) hold significant engineering significance. By quantifying the relationship between the angular correlation error and key engineering parameters such as ADC quantization bits, the major axis amplitude and the standing wave azimuth, the derivation not only provides a quantitative basis for component selection and structural parameter optimization in WA-HRG hardware design but also facilitates the assessment of gyroscope error in practical dynamic scenarios. This enables the targeted design of adaptive error suppression methods to ensure the accuracy of both measurement and control.

### 3.3. Major Axis Amplitude of the Ellipse Error

The HRG characterizes the major axis of the elliptical orbit *a* indirectly through the vibration energy *E*. When quantization error is present, the actual energy $E_{q}$ is expressed as Equation (20):

(20) $$\begin{array}{cc} E_{q} & =c_{x,q}^{2}+s_{x,q}^{2}+c_{y,q}^{2}+s_{y,q}^{2}\\ \; & ={\left(c_{x}+e_{cx}\right)}^{2}+{\left(s_{x}+e_{sx}\right)}^{2}+{\left(c_{y}+e_{cy}\right)}^{2}+{\left(s_{y}+e_{sy}\right)}^{2}\\ \; & \approx E+2(c_{x}e_{cx}+s_{x}e_{sx}+c_{y}e_{cy}+s_{y}e_{sy})\\ \; & =E+\Delta E \end{array}$$

The quadrature vibration is suppressed under ideal conditions, satisfying a≫q [14]. According to Equation (5), the ideal vibration energy *E* can be approximated as Equation (21):

(21) $$E\approx \frac{k_{0}^{2}A^{2}}{4}a^{2}$$

According to Equations (20) and (21), the energy error ΔE and the absolute error of the major axis amplitude Δa are expressed as Equations (22) and (23):

(22) $$\Delta E\approx \frac{k_{0}^{2}A^{2}}{2}a\cdot \Delta a$$

(23) $$\Delta a\approx \frac{2\Delta E}{k_{0}^{2}A^{2}a}=\frac{4(c_{x}e_{cx}+s_{x}e_{sx}+c_{y}e_{cy}+s_{y}e_{sy})}{k_{0}^{2}A^{2}a}\approx \frac{2(\cos2\theta \cdot e_{cx}+\sin2\theta \cdot e_{cy})}{k_{0}A}$$

The relative error of the major axis amplitude is defined as the ratio of the absolute error to the nominal value of the major axis amplitude, as expressed in Equation (24):

(24) $$\Delta a_{\mathrm{rel}}=\frac{\Delta a}{a}\approx \frac{2(\cos2\theta \cdot e_{cx}+\sin2\theta \cdot e_{cy})}{k_{0}Aa}=\frac{C}{k_{0}Aa}\cos\left(2\theta -\varphi_{a}\right)$$

where $C=2\sqrt{{\left(e_{cx}\right)}^{2}+{\left(e_{cy}\right)}^{2}}$ and $\varphi_{a}=\arctan\frac{e_{cy}}{e_{cx}}$.

Analytical derivation indicates that the relative error of the major axis amplitude $\Delta a_{\mathrm{rel}}$ stemming from the quantization process exhibits a negative correlation with ADC quantization bits *n* and the major axis amplitude *a*. Furthermore, this result indicates that even under ideal mechanical conditions, quantization error alone can induce azimuth-dependent amplitude modulation. When the standing wave is positioned at various locations around the hemispherical resonator, quantization error induces a periodic variation in the relative error that tracks the standing wave azimuth θ. The error results in a mismatch between the amplitude of the driving signal and the optimal driving amplitude required to operate the resonator effectively.

### 3.4. Phase Error

The HRG indirectly characterizes the demodulation phase difference δ through the computed parameter *L*. Under ideal conditions, *L* is regulated to zero. In the presence of quantization error, $L_{q}$ is expressed by Equation (25):

(25) $$\begin{array}{cc} L_{q} & =2c_{x,q}s_{x,q}+2c_{y,q}s_{y,q}\\ \; & \approx L+2\left(c_{x}e_{sx}+c_{y}e_{sy}+s_{x}e_{cx}+s_{y}e_{cy}\right)\\ \; & =L+\Delta L\\ \; & =\frac{k_{0}^{2}A^{2}}{4}\left(a^{2}-q^{2}\right)\sin2\left(\delta +\Delta \delta \right) \end{array}$$

Employing the small-angle approximation, i.e., when Δδ≪1, Equation (26) is satisfied:

(26) sin2(δ+Δδ)≈sin2δ+2Δδcos2δ

Combining Equation (25) and (26), the phase error can be derived as Equation (27):

(27) $$\Delta \delta \approx \frac{4(c_{x}e_{sx}+c_{y}e_{sy}+s_{x}e_{cx}+s_{y}e_{cy})}{k_{0}^{2}A^{2}\left(a^{2}-q^{2}\right)\cos2\delta }$$

Under the influence of quantization error, while the measurement and control system remains operational—i.e., δ≈0 and q≪a—Equation (27) can be further simplified to (28):

(28) $$\Delta \delta \approx \frac{2(e_{sx}\cos2\theta +e_{sy}\sin2\theta )}{k_{0}Aa}$$

Analytical derivation indicates that the phase error introduced by the quantization process is negatively correlated with ADC quantization bits *n* and the major axis amplitude *a*. Furthermore, the phase error exhibits significant phase sensitivity characteristics. When cos2δ≈1, Δδ is minimized, thereby allowing the hemispherical resonator to maintain normal vibration. Conversely, when cos2δ≈0, Δδ experiences substantial amplification, which may potentially cause the PLL to lose lock stability. The phase error Δδ consequently induces a phase mismatch between the driving voltage and the vibration phase of the resonator. This mismatch reduces the efficiency of the driving force while simultaneously introducing additional driving force error components into the system.

### 3.5. Driving Force Error

**Figure 5:**
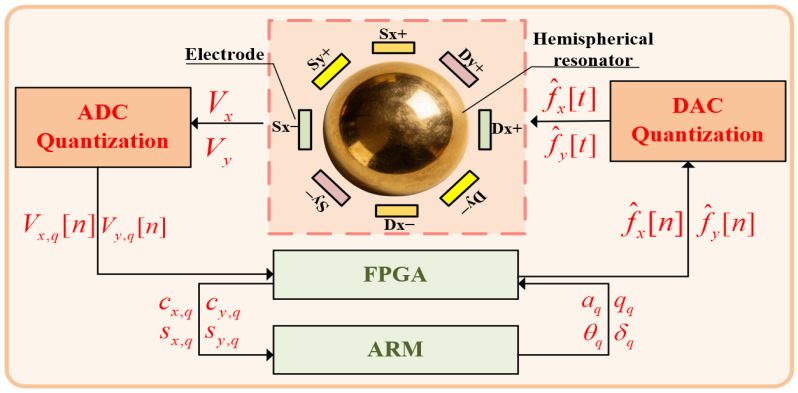
Driving force error transmission mechanism diagram.

The errors of the standing wave azimuth, the major axis amplitude and the phase arising from ADC quantization error are incorporated into the modulation and generation of the driving signal via the control algorithm and closed-loop control system. In addition, as the driving signal undergoes quantization in the DAC, these errors couple with DAC quantization error, collectively transforming into the driving force error acting upon the hemispherical resonator. The mechanism is illustrated in Figure 5.

Based on the driving force Formula (7) and the modeling of ADC quantization error in Section 3.2, Section 3.3 and Section 3.4, the expressions for the actual driving signals are given by Equation (29):

(29) $$\left\{\begin{array}{c} {\hat{f}}_{x}\left[n\right]=f_{x}\left(a_{q},\theta_{q},\delta_{q}\right)=f_{x}\left[n\right]+f_{ex}\left[n\right]\\ {\hat{f}}_{y}\left[n\right]=f_{y}\left(a_{q},\theta_{q},\delta_{q}\right)=f_{y}\left[n\right]+f_{ey}\left[n\right] \end{array}\right.$$

where ${\hat{f}}_{x}\left[n\right]$ and ${\hat{f}}_{y}\left[n\right]$ represent the actual digital driving signals fed into the driving circuit, $f_{x}\left[n\right]$ and $f_{y}\left[n\right]$ represent the ideal driving signals under nominal conditions, and $f_{ex}\left[n\right]$ and $f_{ey}\left[n\right]$ represent the driving error signals introduced due to ADC quantization error.

After DAC, ${\hat{f}}_{x}\left[n\right]$ and ${\hat{f}}_{y}\left[n\right]$ generate the actual driving force applied to the hemispherical resonator, expressed as Equation (30):

(30) $$\left\{\begin{array}{c} {\hat{f}}_{x}\left[t\right]=\left[f_{x}\left[n\right]+f_{ex}\left[n\right]\right]\cdot \mathrm{LSB}\\ {\hat{f}}_{y}\left[t\right]=\left[f_{y}\left[n\right]+f_{ey}\left[n\right]\right]\cdot \mathrm{LSB} \end{array}\right.$$

where ${\hat{f}}_{x}\left[t\right]$ and ${\hat{f}}_{y}\left[t\right]$ represent the actual driving force exerted on the hemispherical resonator, and LSB denotes the least significant bit of the DAC.

## 4. Platform Construction and Simulation Verification

### 4.1. Simulation Platform Construction

**Figure 6:**
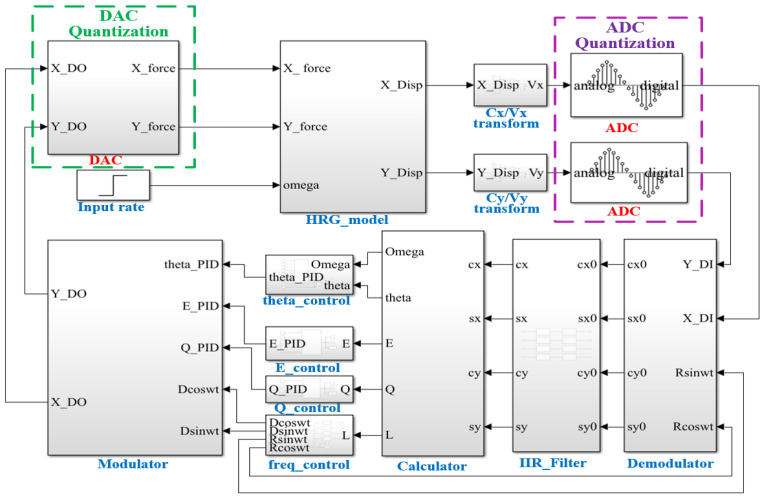
Diagram of the simulation system.

Based on the WA-HRG measurement and control system designed in Section 2.2, a comprehensive closed-loop simulation platform encompassing detection, control and driving functions was constructed, as illustrated in Figure 6.

In the simulation, the initial values of the resonator’s intrinsic characteristic parameters and the measurement and control system parameters are listed in Table 1. These values are representative of a navigation-grade HRG, ensuring the engineering relevance of the simulation.

To investigate and validate the specific factors influencing the errors of standing wave azimuth, the major axis amplitude and the phase in the WA-HRG under the effect of quantization error, this study conducts simulations to analyze the correlations between these errors and the core parameters including the azimuth of the standing wave and the major axis amplitude. At the same time, the impact of quantizers with different bits on the gyroscope’s output is examined. The simulations are carried out from two perspectives: single-parameter validation and multi-parameter collaborative verification. During the simulation process, adjustments to ADC bits, major axis amplitude, and other parameters are made by modifying the program in the SDK. This program is based on the demodulation process in Section 2.3 and defines parameters such as the major axis amplitude and the standing wave azimuth. These definitions effectively reflect the mapping relationships between parameters during signal processing. The program also assigns values to the corresponding SDK scale factors and filtering parameters. In addition, to clearly illustrate the effects of quantization error, the simulation platform retains only quantization errors inherent in the circuit, while other potential error sources such as resonator structural imperfections, electrode assembly errors, and circuit phase errors are deliberately excluded from the model.

### 4.2. Simulation Verification of Angle-Related Errors

**Figure 7:**
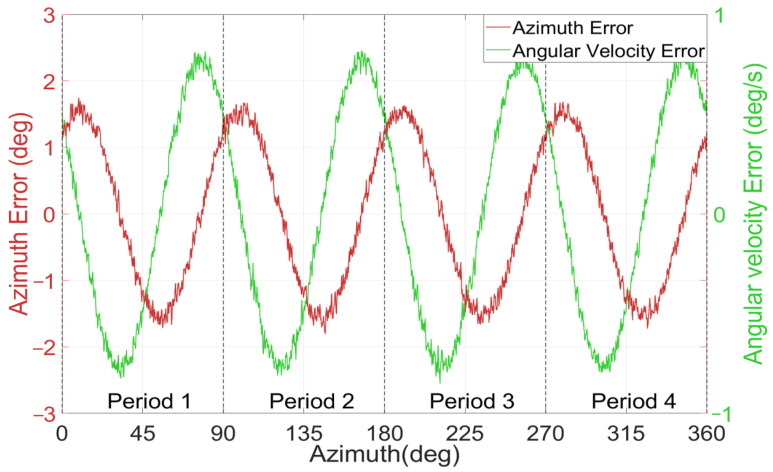
Relationship between angle-related errors and the standing wave azimuth.

Under the condition of 10 ADC quantization bits and an initial major axis amplitude of 10,000 LSB, error curves are plotted according to the azimuth and angular velocity signals output by the gyroscope simulation system and are illustrated in Figure 7. The analysis indicates that over one complete cycle of the azimuth from 0° to 360°, the relationship between both the azimuth errors and angular velocity errors and the azimuth exhibits a distinct fourth-harmonic distribution. The periodicity of these errors is perfectly synchronized, differing only in amplitude and phase. The phase of the angular velocity error leads the phase of the standing wave azimuth error by a constant 90°.

**Figure 8:**
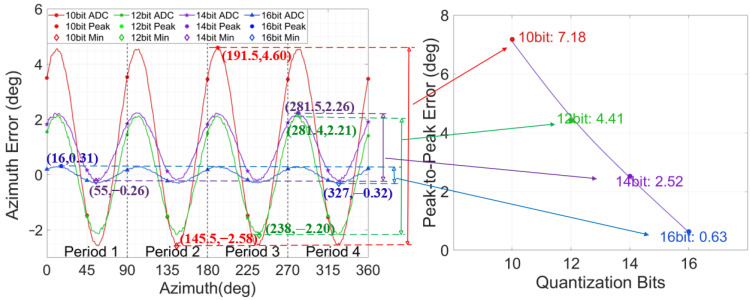
Azimuth Error variation with different ADC quantization bits.

Building upon this, the initial value of the major axis amplitude was reset to 5000 LSB, and the ADC quantization bit was progressively increased from 10 bits to 16 bits. The resulting variation patterns of the azimuth error under different ADC bits are illustrated in Figure 8. The results demonstrate that higher-bit ADC can significantly suppress the azimuth error fluctuations induced by quantization error. In the simulation results, when the ADC quantization bits is 16, the azimuth error caused by quantization is reduced by approximately 91% compared to 10-bit quantization. The magnitude of the error does not strictly follow the variation trend of $\Delta \propto \frac{1}{2^{n}-1}$, which is due to the coupling between the related terms of quantization error and the gyro calculation parameters, consistent with theoretical derivation.

Key data points from the error variation curves are presented in Table 2, further verifying that under different quantization bits, angle-related errors primarily exhibit a fourth-harmonic distribution. The quantization bits primarily affect the fluctuation amplitude of the errors and do not change the fundamental periodic distribution characteristics of the errors. The higher the ADC quantization bits, the smaller the amplitude of the azimuth error. Simultaneously, the azimuth error is not solely determined by the ADC quantization error independently, but is also strongly coupled with the core parameters in the gyroscope’s demodulation and modulation process. This coupling leads to localized non-monotonic characteristics at specific azimuths.

**Figure 9:**
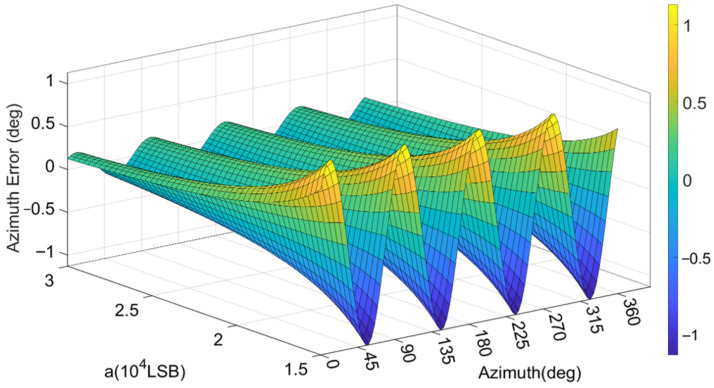
Azimuth error variation with azimuths under different major axis amplitudes.

Further simulation investigating the variation in azimuth error with azimuth under different major axis amplitudes is illustrated in Figure 9. Comprehensive verification indicates that the periodic trend of the azimuth error remains consistent across different amplitudes. The error fluctuation exhibits an inversely proportional decay trend as the major axis amplitude increases.

### 4.3. Simulation Verification of the Major Axis Amplitude Relative Error

**Figure 10:**
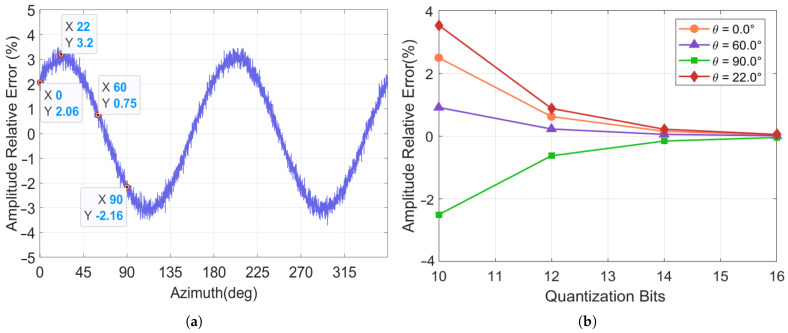
Changes in the amplitude relative error under different (**a**) azimuths and (**b**) quantization bits.

Under the condition of an ADC quantization bit of 10 bits and an initial major axis amplitude of 10,000 LSB, the curve of the amplitude relative error plotted from the amplitude signal output by the gyroscope simulation system is illustrated in Figure 10a. The simulation confirms that as the azimuth advances from 0° to 360°, the relative error of the resonator’s amplitude caused by quantization error induces a periodic variation, with a period of 180°. Subsequently, with the azimuth fixed at 0°, 22°, 60° and 90°, respectively, the ADC quantization bit was progressively increased. The resulting variation patterns of the amplitude relative error under different ADCs with different bits are illustrated in Figure 10b. The figure reveals that the amplitude relative error decays as the quantization bit increases at different azimuths, indicating that higher-bit ADC can significantly suppress the fluctuation in amplitude relative error caused by quantization error.

**Figure 11:**
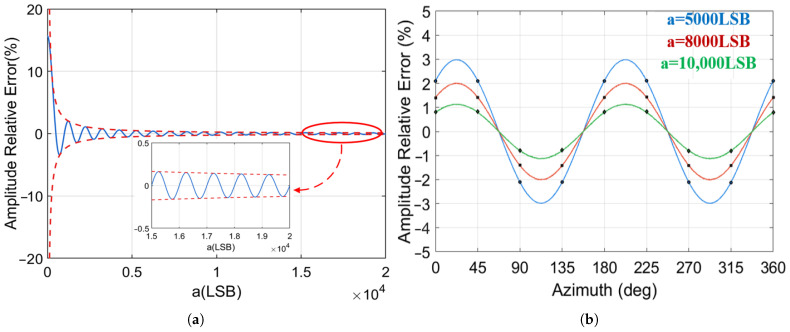
Amplitude relative error analysis: (**a**) Amplitude relative error variation with amplitudes; (**b**) Amplitude relative error variation with azimuths and amplitudes.

The variation patterns of the major axis amplitude relative error under different amplitudes are illustrated in Figure 11a, and its variation with the azimuth is illustrated in Figure 11b. The figures demonstrate that the error exhibits a damped simple harmonic oscillation characteristic across different amplitudes, influenced by the periodic distribution. The oscillation amplitude decays inversely proportionally as the major axis amplitude increases. When the amplitude exceeds 5000 LSB, the error is significantly reduced, which helps suppress relative error and achieve PI control.

### 4.4. Simulation Verification of the Phase Error

Since the analysis method for phase errors is highly similar to that for amplitude errors in Section 4.3, to avoid redundancy, a multi-factor collaborative analysis approach is employed to validate the simulation results in this section.

**Figure 12:**
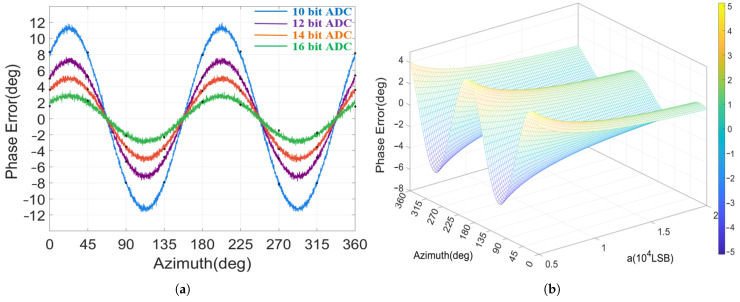
Phase error analysis: (**a**) Phase error variation with azimuths and quantization bits. (**b**) Phase error variation with azimuths and amplitudes.

Under an initial major axis amplitude of 10,000 LSB, the variation in phase error with azimuth across different ADC quantization levels is illustrated in Figure 12a. Subsequently, by altering the major axis amplitudes, the relationship between phase error and both standing wave azimuth and the major axis amplitude is illustrated in Figure 12b. Simulation results indicate that as the azimuth advances from 0° to 360°, the phase error caused by quantization error exhibits a periodic distribution with a 180° cycle. Furthermore, the magnitude of this error diminishes as both the quantization bits and the amplitude increase.

## 5. Conclusions

This paper focuses on quantization error that constrains the high-precision application of the WA-HRG. An in-depth analysis is conducted of the generation mechanisms of quantization error, and theoretical derivation and simulation verification are carried out to assess the impacts of quantization error on key parameter calculations and mode-driven control. This research indicates that under coherent demodulation-based signal processing, the output error caused by quantization error is influenced by numerous factors, including the azimuth of the resonator, the major axis amplitude and the hardware system components. These errors exhibit a periodic distribution that varies with the standing wave azimuth. The angle-related errors show a fourth-harmonic distribution, while the amplitude and phase errors have a period of π.

To suppress error, the standing wave can be processed along the azimuth to reduce differences at different standing wave azimuths, thereby mitigating the periodic fluctuations caused by quantization error. Simultaneously, employing converters with higher quantization bits and increasing the major axis amplitude of the resonator can also significantly reduce the impact of quantization error. Verification in this paper shows that 16-bit ADC quantization bits and an amplitude exceeding 5000 LSB can better match the subtle variations in the HRG resonant signal while also avoiding additional costs and power consumption. This paper provides a clear pathway for subsequent quantization error suppression and compensation, contributing to further improvements in the operational performance of the WA-HRG.

## Figures and Tables

**Table 1:** Initial parameter settings of the simulation platform.

Simulation Parameter	Initial Value
Maximum Resonant Frequency $\omega_{1}$	7000.001 Hz
Minimum Resonant Frequency $\omega_{2}$	7000.000 Hz
Maximum Decay Time Constant $t_{1}$	370.40 s
Minimum Decay Time Constant $t_{2}$	348.80 s
Stiffness Axis Angle $\theta_{\omega }$	4.2 deg
Damping Axis Angle $\theta_{\tau }$	8.7 deg
Initial Electrode Gap $d_{0}$	100 μm
Initial Quadrature Amplitude $q_{0}$	0.0 LSB
Initial Standing Wave Azimuth $\theta_{0}$	0 deg
DC Bias Voltage $V_{dc}$	300 V
Pre-amplifier Circuit Gain $k_{0}$	1.00
Reference Signal Amplitude *A*	2.00
External Rotation Rate Ω	100 deg/s

**Table 2:** Azimuth error under different quantization bits and azimuths.

Azimuth (deg)	Error—10 Bits (deg)	Error—12 Bits (deg)	Error—14 Bits (deg)	Error—16 Bits (deg)
0	3.5110	1.3936	1.8378	0.2024
45	−1.4527	−1.5561	−0.1101	−0.1923
90	3.3977	1.5509	1.8351	0.2428
135	−1.4607	−1.4607	−0.1521	−0.2206
180	3.4999	1.5482	1.8404	0.2013
225	−1.4651	−1.5720	−0.1579	−0.2114
270	3.5238	1.5413	1.7682	0.2041
315	1.4848	−1.5207	−0.1767	−0.1709
360	3.4427	1.4491	1.8887	0.1981

## Data Availability

The data used to support the findings of this study are available from the corresponding author upon request.
